# Tryptophan–Kynurenine Pathway Metabolites in Women Undergoing Caesarean Delivery

**DOI:** 10.3390/life16071166

**Published:** 2026-07-14

**Authors:** Maciej Ziętek, Arkadiusz Bieleninik, Małgorzata Szczuko, Edyta Zagrodnik, Krzysztof Safranow, Marcel Ułan, Bogusław Czerny, Izabela Uzar

**Affiliations:** 1 Department of General Pharmacology and Pharmacoeconomics, Pomeranian Medical University, 71-460 Szczecin, Poland; boguslaw.czerny@pum.edu.pl (B.C.); izabela.uzar@pum.edu.pl (I.U.); 2 Department of Neonatology, Nowy Szpital in Świebodzin, 66-200 Świebodzin, Poland; arkadiuszbieleninik1970@gmail.com; 3 Department of Bromatology and Nutritional Diagnostics, Pomeranian Medical University, 71-460 Szczecin, Poland; malgorzata.szczuko@pum.edu.pl; 4 Clinical Department of Anesthesiology and Intensive Care of Adults and Children, Pomeranian Medical University, 71-460 Szczecin, Poland; edyta.zagrodnik@pum.edu.pl; 5 Department of Biochemistry and Medical Chemistry, Pomeranian Medical University, 71-460 Szczecin, Poland; krzysztof.safranow@pum.edu.pl; 6 Faculty of Medicine, Pomeranian Medical University, 70-204 Szczecin, Poland; marcel.ulan2@gmail.com

**Keywords:** tryptophan, kynurenine, kynurenic acid, quinolinic acid, pregnancy, cesarean section, body mass index

## Abstract

Background: The tryptophan catabolic pathway may play an important role in maternal–fetal immune tolerance, placental function, and metabolic programming, yet its clinical correlates in uncomplicated term pregnancies are not well defined. This study examined how maternal and umbilical artery blood tryptophan metabolites relate to maternal characteristics in women undergoing cesarean section. Methods: In this cross-sectional study, 73 women delivering by cesarean section at a mean gestational age of 38.9 ± 0.96 weeks were enrolled. Maternal venous and umbilical cord arterial blood were collected at delivery, and serum tryptophan (TRP), kynurenine (KYN), kynurenic acid (KYNA), and quinolinic acid (QUIN) were measured using ELISA. Maternal anthropometry, pregnancy complications, antibiotic treatment, and indications for cesarean section were obtained from medical records. Group comparisons used Mann–Whitney U tests, associations were assessed with Spearman’s rank correlations, and selected outcomes were analyzed in multivariable linear regression models. Results: Umbilical concentrations of TRP, KYN, KYNA, and QUIN were higher than the corresponding maternal levels. Maternal TRP was inversely associated with gestational age at delivery and was independently higher in women treated with antibiotics during pregnancy. Cord KYNA correlated positively with pre-pregnancy and pre-delivery BMI, with a stronger association for postpartum BMI, and remained independently associated with pre-delivery BMI in multivariable analysis. Maternal QUIN was higher in women undergoing intrapartum or emergency cesarean section and in those delivered by cesarean section for breech presentation, and both indications were independently associated with higher QUIN in the regression model. No robust associations were observed between TRP, KYN, KYNA, or QUIN and most pregnancy complications, neonatal birth weight, or Apgar scores. Conclusions: Maternal and fetal tryptophan–kynurenine metabolites show distinct, domain-specific relationships with gestational timing, maternal antibiotic exposure during pregnancy, adiposity, and indications or mode of cesarean delivery in term pregnancies. Maternal TRP levels may primarily reflect gestational age and concurrent inflammatory status. Cord blood KYNA may mirror maternal metabolic condition, whereas maternal QUIN appears to be more closely associated with complex obstetric phenotypes than with neonatal status. These exploratory, uncorrected findings suggest that kynurenine pathway metabolites may be associated with selected maternal anthropometric and obstetric factors; none of these associations survived correction for multiple comparisons, and they are strictly hypothesis-generating, requiring confirmation in larger, prospectively designed studies before any biomarker application can be considered. The parallel elevation of the cord KYN/TRP and KYNA/KYN ratios, together with a relative reduction in the QUIN/KYN ratio in the fetal compartment, suggests a metabolic profile characterized by preferential conversion of kynurenine toward the KYNA branch rather than the QUIN branch. This pattern may be consistent with a more neuroprotective metabolic environment in the fetal circulation; however, mechanistic studies are required to confirm this interpretation. The maternal QUIN should be regarded as a marker of obstetric complexity rather than a direct consequence of delivery mode. The study included only women undergoing cesarean delivery; therefore, the findings cannot be generalized to vaginal deliveries.

## 1. Introduction

**Figure 1:**
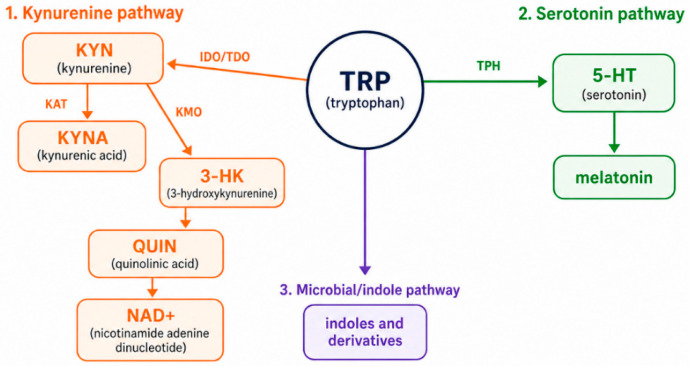
Schematic overview of tryptophan metabolism and its major metabolites during pregnancy. Figure abbreviations: TRP, tryptophan; KYN, kynurenine; KYNA, kynurenic acid; 3-HK, 3-hydroxykynurenine; QUIN, quinolinic acid; NAD+, nicotinamide adenine dinucleotide; 5-HT, 5-hydroxytryptamine; IDO, indoleamine 2,3-dioxygenase; TDO, tryptophan 2,3-dioxygenase; KAT, kynurenine aminotransferase; KMO, kynurenine 3-monooxygenase; TPH, tryptophan hydroxylase.

Pregnancy is a highly dynamic physiological state in which maternal metabolism, immune adaptation, and placental function are continuously adjusted to support fetal development. This process requires a finely regulated balance between immune tolerance and defense, nutrient allocation, endocrine signaling, and vascular remodeling. Among the biochemical pathways involved in these adaptations, tryptophan metabolism has emerged as one of the most biologically relevant, because it links maternal nutritional status with immune regulation, placental homeostasis, and fetal programming. Tryptophan is an essential amino acid that cannot be synthesized de novo in humans and therefore depends on maternal intake and systemic availability [1]. Current evidence suggests that the fetus is unlikely to be a major source of de novo tryptophan synthesis; rather, fetal tryptophan availability depends predominantly on maternal supply and placental transport and metabolism [2]. In pregnancy, its metabolism is redirected through several pathways, with the kynurenine pathway representing the principal route of catabolism. The kynurenine pathway generates a series of biologically active metabolites, including kynurenine, kynurenic acid (KYNA), and quinolinic acid (QUIN), each of which may exert distinct physiological effects (Figure 1).

KYNA is generally considered a neuroactive and immunomodulatory metabolite with potentially protective properties, whereas QUIN is more often associated with pro-inflammatory, oxidative, and neurotoxic effects [1,3]. These metabolites are not merely by-products of tryptophan degradation; rather, they function as signaling molecules that may influence immune responses, vascular tone, oxidative balance, and cellular metabolism [3,4]. In this way, the kynurenine pathway can be viewed as a metabolic interface integrating maternal physiology, placental function, and fetal development. A particularly important site of tryptophan metabolism during pregnancy is the placenta, which dynamically regulates fetal exposure to serotonin and kynurenine pathway metabolites [5]. The placenta expresses key enzymes involved in the kynurenine pathway, including indoleamine 2,3-dioxygenase 1, which catalyzes the first and rate-limiting step of tryptophan degradation. By modulating local tryptophan availability and producing kynurenine metabolites, the placenta contributes to the maintenance of maternal–fetal immune tolerance. This is essential because the fetus represents a semi-allogeneic graft that must be tolerated by the maternal immune system while still allowing adequate host defense. Placental tryptophan metabolism is also implicated in trophoblast function, angiogenesis, and the regulation of inflammatory pathways, all of which are critical for normal placental development and pregnancy progression [6]. Growing evidence indicates that disturbances in tryptophan metabolism may accompany or contribute to adverse pregnancy outcomes [7]. Altered levels of tryptophan and its metabolites have been reported in association with preeclampsia, fetal growth abnormalities, preterm birth, gestational metabolic disturbances, and inflammatory conditions [7,8]. Because the kynurenine pathway is sensitive to immune activation, oxidative stress, and metabolic state, it may reflect both physiological adaptation and pathological dysregulation during pregnancy. This makes tryptophan metabolites attractive candidates for biomarker research, especially in studies aiming to characterize the maternal–fetal interface more precisely. Maternal metabolic phenotype appears to be particularly important in this context. Obesity, excessive gestational weight gain, insulin resistance, and chronic low-grade inflammation may all influence tryptophan catabolism and the balance of downstream metabolites [7,9,10]. Previous studies have shown that maternal adiposity can be associated with altered cord blood concentrations of kynurenine pathway metabolites, suggesting that maternal metabolic status shapes fetal exposure to these compounds [7,11]. Such findings are consistent with the concept of developmental programming, in which intrauterine metabolic and inflammatory signals may influence later offspring health [12]. In this regard, KYNA and other kynurenine metabolites may serve not only as biomarkers of current maternal physiology but also as indicators of the fetal metabolic environment [13]. In addition to maternal adiposity, pregnancy complications and clinical exposures may modify the kynurenine pathway. Infectious episodes, antibiotic use, operative delivery, and obstetric interventions are all potential markers of altered inflammatory or metabolic states [14,15]. Antibiotic therapy in pregnancy often reflects an underlying infection or inflammatory process rather than a pharmacological effect alone, and such processes may influence tryptophan metabolism through immune-mediated activation of the kynurenine pathway [16]. Likewise, cesarean delivery may be associated with distinct metabolic and inflammatory patterns compared with vaginal birth, potentially reflecting differences in maternal stress response, fetal condition, and the overall obstetric context [17]. These associations highlight the need to interpret tryptophan metabolites in relation to the clinical background of pregnancy rather than in isolation. Of note, the direction and magnitude of changes in tryptophan metabolism may depend on gestational age [18]. Early and late pregnancy are characterized by different hormonal, immune, and placental environments, and the activity of the kynurenine pathway appears to change across gestation [19]. This temporal variability is important because it suggests that the same metabolite may have different biological significance depending on the stage of pregnancy in which it is measured. Therefore, the interpretation of maternal or cord blood concentrations requires careful attention to the timing of sampling, gestational age, and perinatal circumstances. Although interest in tryptophan metabolism in pregnancy has increased substantially in recent years, the clinical significance of individual metabolites remains incompletely understood [20,21]. Available studies are still relatively limited, often include small cohorts, and use heterogeneous analytical approaches, which makes direct comparison difficult. Moreover, many studies focus on isolated clinical outcomes rather than integrating maternal metabolic parameters, obstetric history, and neonatal exposure within one framework. There is therefore a need for further investigations that examine tryptophan metabolites in relation to a broader clinical phenotype of pregnancy. A deeper understanding of this pathway may have several implications. First, it may improve insight into the biological mechanisms underlying pregnancy adaptation and maladaptation. Second, it may help identify biomarkers that capture both maternal metabolic health and placental function. Third, it may support future risk stratification in obstetrics by identifying metabolomic patterns associated with specific pregnancy characteristics or complications. Ultimately, such knowledge could contribute to more individualized prenatal assessment and improved maternal–fetal care.

In the present study, we evaluated associations between selected maternal and cord blood tryptophan metabolites and clinical parameters of pregnancy and delivery. We specifically focused on relationships with gestational age, maternal metabolic status, antibiotic exposure, and mode of delivery, aiming to better characterize the biological and clinical relevance of the kynurenine pathway in pregnancy. We hypothesized that distinct metabolites would reflect different aspects of maternal–fetal physiology, including placental adaptation, inflammatory exposure, and perinatal outcome.

## 2. Materials and Methods

### 2.1. Study Group

A total of 96 women scheduled for cesarean delivery were assessed for eligibility. Of these, 23 women were excluded for the following reasons: lack of informed consent (n = 9), twin pregnancy (n = 6), and incomplete clinical or laboratory data (n = 8). Finally, 73 women with singleton term pregnancies who delivered by cesarean section and had complete clinical, obstetric, and neonatal data available were included in the final analysis. Only women who delivered by cesarean section were included in the present study in order to obtain a clinically homogeneous cohort and to minimize confounding related to the mode and timing of delivery. Cesarean delivery is associated with characteristic maternal hemodynamic, endocrine, inflammatory, and anesthetic conditions that differ substantially from those observed in vaginal birth, and these factors may independently influence both maternal and neonatal biochemical and clinical parameters. Restricting the study population to cesarean sections therefore reduced heterogeneity arising from variable lengths of labor, differing degrees of uterine activity, and variations in analgesia or anesthesia techniques typically encountered in vaginal deliveries. Moreover, the standardized perioperative management protocols used for cesarean sections at our institution facilitated more reliable comparison of outcomes within the cohort. This approach increased the internal validity of the analyses and enabled a more precise assessment of the relationships between the investigated variables and maternal–fetal condition in the specific context of cesarean delivery.

**Figure 2:**
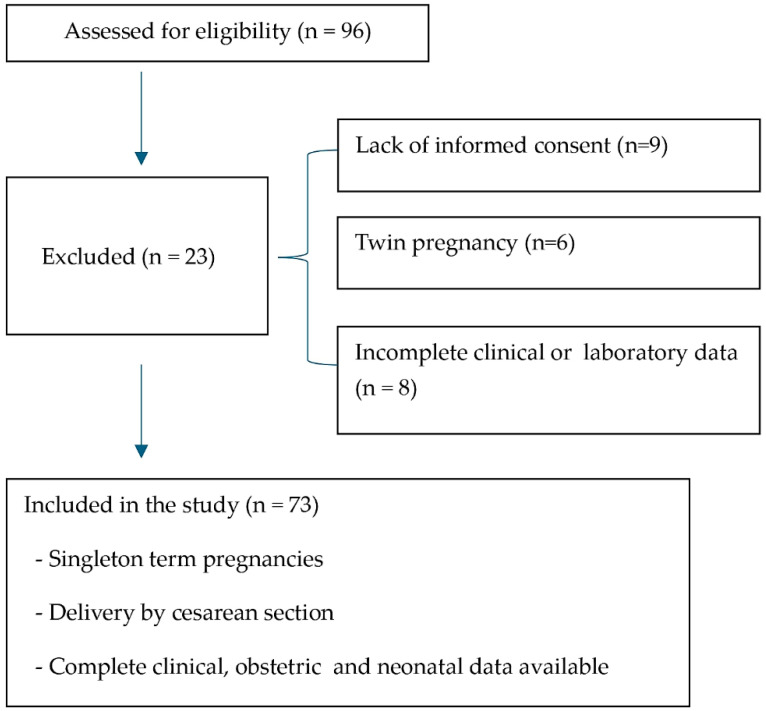
Patient selection scheme for the study.

The patient selection process for the study is presented below Figure 2. Clinical and obstetric data were retrieved from medical records. The analyzed cohort comprised women with singleton pregnancies delivered at a mean gestational age of 38.89 ± 0.96 weeks. The mean maternal height was 1.67 ± 0.06 m. The mean pre-delivery body weight and body mass index (BMI) were 79.20 ± 20.79 kg and 28.18 ± 6.92 kg/m^2^, respectively, while in the early pre-delivery period the corresponding values were 92.04 ± 19.82 kg and 32.78 ± 6.44 kg/m^2^. The gestational weight gain implied by the difference between pre-pregnancy and pre-delivery weight (mean 12.84 kg) is consistent with expected physiological weight gain during pregnancy. No independently measured true postpartum (post-cesarean) weight was collected in this cohort, and that the main analysis instead uses pre-delivery BMI, obtained prior to cord blood sampling. Maternal body mass index (BMI) was classified according to WHO criteria as underweight (BMI < 18.5 kg/m^2^), normal weight (BMI 18.5–24.9 kg/m^2^), overweight (BMI 25.0–29.9 kg/m^2^), and obese (BMI ≥ 30.0 kg/m^2^). Maternal demographic, anthropometric, obstetric, and neonatal data were collected for further analysis (Table 1). The mean neonatal birth weight was 3492.74 ± 444.76 g. Among the 73 neonates included in the study, overall birth outcomes were favorable, reflected by high Apgar scores at all assessed time points. The mean Apgar score at 1 min was 9.58 ± 0.82, with a median of 10 and a range of 6 to 10, indicating that the vast majority of newborns were in good condition immediately after birth, with only a few requiring closer initial observation. By 3 min, the mean Apgar score increased to 9.75 ± 0.55, with a median of 10 and a range of 8 to 10, demonstrating rapid postnatal adaptation and effective early neonatal management. At 5 min, neonatal status remained good, with a mean Apgar score of 9.85 ± 0.40, a median of 10, and a range of 8 to 10, confirming sustained good clinical condition in nearly all infants in this cohort.

### 2.2. Recruitment and Eligibility

Women were recruited at the Department of Perinatology, Obstetrics and Gynaecology, Pomeranian Medical University in Szczecin, Poland, between January 2024 and December 2024. Eligible participants were women undergoing cesarean delivery who provided informed consent for participation in the study and use of their clinical data and biological material, if applicable.

### 2.3. Timing and Mode of Recruitment

Participants were recruited before delivery during hospitalization before planned or emergency cesarean section. In cases of urgent cesarean section, inclusion in the study and consent procedures were conducted only if the patient’s clinical condition allowed for informed and voluntary participation and did not interfere with standard obstetric management. No study-related procedures delayed emergency clinical care.

### 2.4. Clinical Data Collection

Maternal and neonatal clinical data were obtained from hospital records. Maternal age, height, body weight, body mass index (BMI), gestational age at delivery, number of previous cesarean sections, indication for cesarean section, neonatal birth weight, and Apgar scores were recorded. Pre-pregnancy body weight was self-reported by the patient, based on recalled body weight prior to conception. Pre-delivery body weight was measured by clinical staff at hospital admission on the day of cesarean delivery. Pre-pregnancy and pre-delivery BMI were calculated from these respective weights and maternal height. Pre-delivery BMI was categorized using standard WHO adult thresholds (normal weight: 18.5–24.9 kg/m^2^; overweight: 25.0–29.9 kg/m^2^; obese: ≥30.0 kg/m^2^).

In this cohort, the most frequent indication for cesarean section was cephalopelvic disproportion, accounting for nearly one fifth of all procedures. Tokophobia, reflecting maternal request driven by fear of vaginal birth, represented the second most common indication. Breech presentation was also a relevant obstetric reason for surgery, although less frequent than cephalopelvic disproportion and tokophobia. Among non-obstetric causes, ophthalmologic and orthopedic indications together constituted a smaller but clinically important proportion of cesarean deliveries (Table 2).

### 2.5. Sample Collection and Biochemical Analyses

Maternal and umbilical artery blood samples were collected in the delivery suite as part of routine intrapartum procedures. Maternal venous blood was obtained on the day of delivery during standard blood sampling in the labor ward and collected into serum tubes. Blood samples were collected into standard 7.5 mL serum tubes (S-Monovette^®^, Sarstedt, Nümbrecht, Germany). After clotting for 10–20 min at room temperature, samples were centrifuged at 530–1200× *g* (corresponding to 2.000–3.000 rpm; fixed-angle rotor 12 × 15/10 mL, rotor radius 11.9 cm, angle 30°; MPW-251, MPW Med. Instruments Workers’ Cooperative, Boremlowska 46, 04-347 Warsaw, Poland) for 20 min at room temperature (20–22 °C), and the serum supernatant was carefully transferred into labeled polypropylene tubesand stored at −80 °C until analysis. Umbilical cord blood was collected immediately after delivery of the newborn during cesarean section; blood was drawn from the umbilical artery into serum tubes before placental separation, allowed to clot for 10–20 min at room temperature, centrifuged under identical conditions (530–1200× *g*, 20 min, 20–22 °C, same fixed-angle rotor), and the resulting serum was aliquoted and stored at −80 °C until batch analysis.

Maternal and cord blood concentrations of tryptophan, kynurenine, kynurenic acid and quinolinic acid were measured using commercially available enzyme-linked immunosorbent assay (ELISA) kits, according to the manufacturers’ instructions.

Kynurenic acid concentrations were measured using a commercial sandwich enzyme-linked immunosorbent assay (Human Kynurenic Acid ELISA Kit, Cat. No. E3982Hu, Bioassay Technology Laboratory, Shanghai, China), designed for quantitative determination of KYNA in serum, plasma and other biological fluids. The assay features a standard curve range of 0.5–150 nmol/L and a manufacturer-reported sensitivity of 0.21 nmol/L, with intra- and inter-assay coefficients of variation of approximately 8% and 10%, respectively. Tryptophan concentrations were determined using a sandwich ELISA (Human Tryptophan ELISA Kit, Cat. No. E4244hu, Bioassay Technology Laboratory, Shanghai, China) intended for quantitative measurement of tryptophan in serum, plasma and other biological matrices. According to the manufacturer, the assay has a measuring range of 0.5–200 µg/mL and a sensitivity of 0.23 µg/mL, with intra- and inter-assay coefficients of variation of about 8% and 10%, respectively. Kynurenine concentrations were assessed using a competitive ELISA (Human Kynurenine ELISA Kit, Cat. No. EA0098Hu, Bioassay Technology Laboratory, Shanghai, China) dedicated to the quantification of KYN in serum, plasma, cell culture supernatants and other biological fluids. The declared assay range is 3–900 pmol/mL, with a sensitivity of 1.24 pmol/mL, and intra- and inter-assay coefficients of variation not exceeding 10% and 12%, respectively, according to the manufacturer’s specifications. Quinolinic acid concentrations were measured using a commercial competitive ELISA (All species Quinolinic Acid ELISA Kit, Competitive EIA, Cat. No. LS-F25110, LSBio, Shirley, MA, USA) intended for quantitative determination of QA in plasma and serum from various species under experimental conditions. The manufacturer reports a detection range of 1.23–100 ng/mL and a typical sensitivity of <0.55 ng/mL, with intra- and inter-assay coefficients of variation below 10% and 12%, respectively.

On the day of analysis, frozen serum aliquots were thawed on ice, gently mixed, and, when necessary, diluted with the appropriate standard/sample diluent (typically 1:2 to1:5) to fall within the linear range of each assay. For tryptophan and kynurenic acid, sandwich ELISA kits were used: pre-coated wells with specific antibodies captured the analyte, followed by incubation with biotinylated detection antibodies and streptavidin–horseradish peroxidase (HRP). After a 60 min incubation at 37 °C, plates were washed five times with 1× wash buffer, substrate solutions were added and incubated for 10 min at 37 °C in the dark, and the reaction was stopped with acidic stop solution.

Kynurenine was measured using a competitive ELISA kit, in which endogenous kynurenine in the sample competed with a biotinylated antigen for binding sites. Standards and diluted samples were incubated with biotinylated antigen for 60 min at 37 °C, followed by washing and a further 60 min incubation with avidin-HRP. After washing, chromogenic substrate was added, the reaction was stopped, and signal intensity, measured at 450 nm, was inversely proportional to kynurenine concentration. Quinolinic acid concentrations in maternal and cord serum were determined using a dedicated ELISA-based kit following the same general workflow: addition of standards and samples to pre-coated wells, incubation with the corresponding detection system, washing, substrate development, and spectrophotometric reading at 450 nm. For all assays, standard curves were generated for each plate, and analyte concentrations were interpolated from the calibration curves with correction for the applied dilution factor.

Owing to constraints on available sample volume, all samples were analysed in singlet rather than in duplicate. Tryptophan (TRP) and kynurenine (KYN) concentrations were measured across six assay plates; kynurenic acid (KYNA) and quinolinic acid (QUIN) were measured across five plates. A single reference quality-control (QC) sample was included on each plate; the between-run coefficient of variation (CV) for the QC sample was approximately 9–12%, depending on the analyte. Maternal and umbilical cord blood samples from the same mother–neonate dyad were analysed on the same plate where logistically possible, to minimise inter-plate variability in paired comparisons; this was not achieved for all dyads. Formal assessments of dilution linearity, recovery (spike-in experiments), within-run replicate CV, and outlier-handling procedures beyond the exclusion of values outside the manufacturer’s declared detection range were not performed in this study. These analytical steps, along with the use of commercially available ELISA kits rather than liquid chromatography-tandem mass spectrometry (LC-MS/MS), represent important methodological limitations. LC-MS/MS is the current reference standard for kynurenine pathway metabolite quantification in biological fluids and offers higher specificity and lower susceptibility to antibody cross-reactivity than ELISA; it was not available at our institution at the time of sample analysis. The ELISA method was selected as the only feasible approach given local laboratory infrastructure. Accordingly, the absolute concentration values reported here should not be compared directly to those from LC-MS/MS-based studies in the literature; cross-study comparisons are restricted to the direction and relative pattern of associations rather than absolute values.

### 2.6. Statistical Analysis

Statistical analyses were performed using nonparametric methods because the distributions of the continuous variables were asymmetric and/or deviated from normality. Descriptive statistics are presented as mean ± standard deviation (SD), median, minimum and maximum values, and interquartile range (Q1–Q3) for continuous variables, and as counts and percentages for categorical variables. Group comparisons for continuous variables were carried out using the Mann–Whitney U test, with results reported as exact two-sided *p*-values. Associations between continuous biochemical parameters and clinical variables (including maternal and umbilical tryptophan-pathway metabolites, maternal anthropometric measures, and neonatal outcomes) were assessed using Spearman’s rank correlation coefficient (rho). General linear model (GLM) was used for multivariable analysis to find independent factors associated with the studied metabolite concentrations. Only independent variables significantly associated with the dependent variable both in the univariate analysis and after inclusion into the model were selected. All statistical tests were two-tailed, and a *p*-value < 0.05 without correction for multiple comparisons was considered statistically significant. Continuous variables are presented as median with interquartile range (IQR: 25th–75th percentile). Paired comparisons between maternal peripheral blood and umbilical cord blood concentrations of tryptophan (TRP), kynurenine (KYN), kynurenic acid (KYNA), and quinolinic acid (QUIN) were performed using the Wilcoxon signed-rank test. This test was applied to the subset of patients (n = 73) for whom simultaneous paired maternal and cord blood samples were available. Metabolic activity indices of the kynurenine pathway were calculated as molar concentration ratios: KYN/TRP (reflecting IDO/TDO enzymatic activity), KYNA/KYN (reflecting kynurenine aminotransferase, -KAT-activity), and QUIN/KYN (reflecting the activity of the quinolinate-producing branch). Cord-to-maternal ratios of these indices were subsequently derived to quantify the relative enrichment of each metabolic index in the fetal compartment. The strength and direction of associations between paired maternal and cord blood values were assessed using Spearman’s rank correlation coefficient (r_s). Correlations were considered statistically significant at a two-tailed *p*-value < 0.05. All statistical analyses were performed using commercially available software Statistica, version 12.5 (StatSoft Inc., Tulsa, OK, USA).

## 3. Results

Maternal and umbilical concentrations of tryptophan pathway metabolites are summarized in Table 3. Tryptophan (TRP): concentrations expressed in micrograms per milliliter (µg/mL), according to the working range of the tryptophan ELISA. Kynurenic acid (KYNA): concentrations expressed in nanomoles per liter (nmol/L), consistent with the KYNA ELISA standard curve. Kynurenine (KYN): concentrations expressed in picomoles per milliliter (pmol/mL), in line with the declared assay range. Quinolinic acid (QUIN): concentrations expressed in nanograms per milliliter (ng/mL), reflecting the detection range of the quinolinic acid ELISA. Overall, umbilical levels of TRP, KYNA, KYN, and QUIN were higher than the corresponding maternal concentrations, with relatively wide ranges for all metabolites. Mean maternal TRP, KYNA, KYN, and QUIN values were 5487.17 ± 1397.97, 4.27 ± 1.49, 285.33 ± 112.17, and 47.85 ± 15.18, respectively, whereas the mean umbilical values reached 11,738.47 ± 2302.51, 63.36 ± 16.54, 1025.07 ± 240.58, and 130.30 ± 31.21, respectively.

Table 4 shows that, among all clinical subgroupings examined, only two comparisons yielded statistically significant differences in metabolite concentrations.

Among the 73 women who delivered by cesarean section, statistically significant differences in maternal blood tryptophan metabolites were observed between those undergoing elective procedures (n = 63) and those who had cesarean delivery during labor or as an emergency (n = 10). In umbilical cord blood, significant differences in metabolite concentrations were also noted between neonates of women with normal postpartum weight (n = 50) and those of women who were overweight or obese after delivery (n = 23).

Maternal quinolinic acid levels were higher in women undergoing intrapartum or emergency cesarean section than in those scheduled for elective procedures, suggesting that more acute or stressful intrapartum conditions may be associated with activation of the kynurenine pathway toward quinolinic acid production. At the same time, umbilical kynurenic acid concentrations were higher in neonates born to women with postpartum overweight or obesity compared with those without excessive postpartum weight, indicating a potential link between maternal adiposity in the peripartum period and increased fetal/umbilical exposure to kynurenic acid. For all other clinical factors (including gestational diabetes, hypertension, thyroid dysfunction, antibiotic therapy, and detailed indications for cesarean section), no significant between-group differences in maternal or umbilical metabolite levels were detected, suggesting that their influence, if present is smaller than the effects of delivery mode and postpartum adiposity in this cohort. Statistically insignificant data are presented in Supplementary Materials (Table S1).

Table 5 summarizes only those clinical-biochemical associations that reached statistical significance in correlation analyses. Higher umbilical KYNA concentrations correlated positively with birth weight and postpartum BMI, which is consistent with the group comparisons and further supports a relationship between greater fetal growth/maternal adiposity and increased kynurenic acid levels in the umbilical circulation. In contrast, higher maternal QUIN and TRP concentrations were associated with lower Apgar scores at 1 min, and higher maternal TRP also correlated inversely with Apgar at 5 min, suggesting that elevated maternal tryptophan-pathway activity around delivery may be linked to less favorable immediate neonatal adaptation. The absence of additional significant correlations for other clinical variables implies that, in this sample, the strongest and most consistent associations involve early neonatal condition (Apgar scores) and indices of fetal growth or maternal body mass, rather than maternal age or routine anthropometric measures alone. The observed correlations between Apgar scores and maternal tryptophan-pathway metabolites are now explicitly identified as not withstanding correction for multiple comparisons, and are therefore reported as exploratory associations that do not meet the threshold of statistical significance following adjustment for the false discovery rate.

Three general linear models (GLM) were constructed to explore independent predictors of selected tryptophan-pathway metabolites, and the detailed parameter estimates are presented in Table 6, Table 7 and Table 8.

In Table 6, the univariate linear model for umbilical kynurenic acid with postpartum BMI as the only predictor demonstrates a significant positive association. No other significant independent predictor for umbilical kynurenic acid was found. Each one-unit increase in postpartum BMI was associated with an increase in umbilical KYNA concentration of roughly 0.8 ng/mL. The model explains a relevant proportion of variability in umbilical KYNA (R^2^ = 0.09), indicating that higher maternal adiposity in the early postpartum period is independently linked to elevated kynurenic acid levels in umbilical blood.

Table 7 presents the multivariable linear model for maternal tryptophan (R^2^ = 0.17), which included gestational age at delivery, antibiotic treatment during pregnancy, and Apgar score at 1 min. In this model, antibiotic use during pregnancy emerged as a significant positive predictor of maternal TRP concentrations, with women exposed to antibiotics showing substantially higher maternal TRP levels (*p* ≈ 0.02). Gestational age showed a negative association with maternal TRP and Apgar score at 1 min also tended to correlate inversely with maternal TRP, both effects being of borderline statistical significance (*p* around 0.05–0.07). Overall, the results summarized in Table 3, Table 4 and Table 5 suggest that maternal tryptophan-pathway activity near term is modulated not only by delivery-related factors and neonatal condition, but also by maternal adiposity and perinatal antibiotic exposure.

Table 8 presents the multivariable linear regression model for maternal quinolinic acid concentration, including intrapartum/emergency cesarean section and breech presentation as an indication for cesarean section as independent variables. Intrapartum/emergency cesarean section was significantly associated with higher maternal QUIN compared with elective cesarean section. Breech presentation was also significantly associated with higher maternal QUIN. The overall model was statistically significant, with multiple R = 0.375, R^2^ = 0.141, and adjusted R^2^ = 0.116. This pattern indicates that maternal QUIN is modestly but independently higher in women with more complex obstetric courses, rather than being uniformly elevated across all cesarean deliveries. In particular, the standardized coefficients suggest that breech presentation has a slightly stronger association with maternal QUIN than intrapartum/emergency cesarean section, although both effects are of similar magnitude. Given that the model explains approximately 14% of the variance in maternal QUIN concentrations, these indications appear to contribute meaningfully to maternal QUIN variability but do not fully account for it, implying that additional clinical or biological factors are likely involved.

Table 9 shows an analysis of 73 paired maternal-umbilical cord blood samples and demonstrates that all four kynurenine pathway metabolites: TRP, KYN, KYNA, and QUIN were significantly elevated in cord blood compared to maternal blood (Wilcoxon signed-rank test, all *p* < 0.0001). Despite these quantitative differences, maternal and cord blood concentrations were largely not correlated (Spearman r non-significant for TRP, KYN, and KYNA), indicating that fetal metabolite levels are regulated independently of the maternal compartment, rather than reflecting simple transplacental equilibration Table 9.

**Figure 3:**
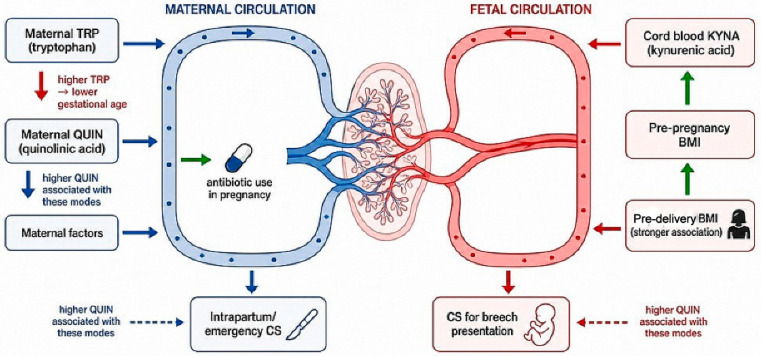
Uncorrected exploratory associations between maternal and cord blood tryptophan–kynurenine metabolites and selected clinical characteristics.

The main associations between maternal and cord blood tryptophan–kynurenine metabolites and clinical characteristics are summarised in a schematic illustration (Figure 3). The diagram does not imply causality or represent a validated biological pathway model; all depicted associations are exploratory and require independent replication. Figure 3 illustrates the main relationships identified between maternal circulation (blue) and fetal/umbilical circulation (red) for tryptophan (TRP), kynurenine (KYN), kynurenic acid (KYNA), and quinolinic acid (QUIN). Solid arrows indicate statistically significant associations. In the maternal compartment, the red downward arrow from maternal TRP denotes an inverse association between higher maternal TRP concentrations and gestational age at delivery (higher TRP associated with lower gestational age). Solid blue arrows from maternal QUIN toward intrapartum/emergency cesarean section and maternal factors indicate higher maternal QUIN levels in women undergoing intrapartum or emergency cesarean section and in those delivered by cesarean section for breech presentation. In the fetal compartment, solid green arrows from pre-pregnancy BMI and postpartum BMI to cord blood KYNA depict positive associations, with a stronger relationship for postpartum BMI, reflecting higher cord KYNA concentrations in offspring of women with greater postpartum adiposity. Red arrows on the fetal side highlight that these KYNA differences are observed in umbilical cord blood. Dashed arrows represent additional significant but more context-dependent associations, emphasizing that higher maternal QUIN is particularly linked to intrapartum/emergency cesarean section and cesarean section for breech presentation rather than to all cesarean deliveries uniformly. TRP, tryptophan; KYN, kynurenine; KYNA, kynurenic acid; QUIN, quinolinic acid; BMI, body mass index; CS, cesarean section. Antibiotic exposure was included in the analysis as a clinical marker extracted from medical records and was not interpreted as a direct determinant of tryptophan metabolism. Antibiotics were administered during pregnancy in different trimesters, typically for at least 7 consecutive days, most commonly due to upper respiratory tract infections or urinary tract infections. In this context, antibiotic treatment is considered to reflect underlying infectious or inflammatory processes rather than a pharmacological effect of the drugs themselves on the tryptophan–kynurenine pathway. The associations depicted are unadjusted for multiple comparisons do not imply causality, and should not be interpreted as an integrated biological pathway model.

## 4. Discussion

The present findings suggest that maternal and fetal tryptophan–kynurenine metabolism is tightly linked to gestational timing, maternal infectious/inflammatory burden as reflected by antibiotic treatment, maternal adiposity, and mode of operative delivery. In this cohort, higher maternal tryptophan (TRP) was associated with lower gestational age at delivery and with a history of antibiotic therapy during pregnancy; however, antibiotic exposure was analyzed as a clinical marker extracted from medical records and most often reflected episodes of upper respiratory tract or urinary tract infection treated for at least 7 days in different trimesters, rather than a direct pharmacological modifier of TRP metabolism. In addition, higher cord blood kynurenic acid (KYNA) was associated with higher pre-pregnancy and pre-delivery BMI, with a stronger relationship for pre-delivery BMI, and higher maternal quinolinic acid (QUIN) was associated with cesarean delivery, particularly cesarean section performed because of breech presentation. At the same time, only modest inverse correlations were observed between maternal TRP or QUIN and early Apgar scores, and no robust associations were noted between TRP, KYNA or QUIN and most pregnancy complications, indicating a certain specificity of these relationships rather than a global link with all perinatal outcomes. These observations are biologically plausible given the central role of the placental kynurenine pathway in immune tolerance, vascular regulation, oxidative balance, and fetal metabolic programming [5,10].

### 4.1. Maternal TRP and Gestational Age

The inverse association between maternal TRP and gestational age may reflect the dynamic regulation of the placental tryptophan–kynurenine axis across pregnancy. The placenta expresses indoleamine 2,3-dioxygenase 1 (IDO1) and other kynurenine-pathway enzymes in a gestational-age-dependent manner, and placental kynurenine metabolism increases as pregnancy advances, supporting local immunoregulation, vascular adaptation and fetal supply of neuroactive metabolites [18]. In this context, higher maternal TRP at lower gestational age may indicate an earlier stage of placental metabolic adaptation, before progressive substrate utilization and downstream catabolism intensify toward term [5]. This pattern is also consistent with the concept that circulating TRP does not simply represent dietary availability, but is shaped by placental transport, immune activation, and metabolic demand [19,20]. Because maternal and fetal TRP pools are interconnected through active placental transport, variations in maternal TRP likely reflect both maternal physiology and placental function. Thus, gestational age should be treated as a major determinant when interpreting maternal tryptophan-related metabolites in pregnancy studies [10].

### 4.2. Antibiotic Exposure and TRP

The association between higher maternal TRP and antibiotic use during pregnancy is most likely indirect and may reflect infection-related modulation of the kynurenine pathway rather than a direct effect of antibiotics themselves. Antibiotic treatment during pregnancy usually marks intercurrent infection or inflammatory complications, both of which can alter tryptophan metabolism through immune activation and changes in IDO1 activity. This interpretation is strengthened by the known sensitivity of placental kynurenine metabolism to inflammatory cues and pregnancy complications [5,20]. This interpretation is also supported by experimental and clinical observations indicating that maternal immune activation and antibiotic exposure may modify tryptophan catabolism and downstream metabolite profiles. From a biological perspective, infection-driven immune activation can shift tryptophan flux toward kynurenine metabolites, yet the direction and magnitude of change may vary depending on the severity, timing, and localization of inflammation. Therefore, antibiotic exposure in our dataset is best considered a surrogate marker of a broader inflammatory milieu rather than a causal driver of TRP elevation. This point supports the idea that maternal TRP may be viewed as a sensitive readout of pregnancy stressors, including infection, inflammation, and placental adaptation, rather than as a marker of drug exposure per se [10,14].

### 4.3. Cord KYNA and Maternal BMI

The positive association between cord blood KYNA and maternal BMI before and after pregnancy fits well with reports that maternal obesity is linked to altered kynurenine metabolism in both maternal and fetal compartments [13]. In this cohort, cord KYNA correlated with both pre-pregnancy and pre-delivery BMI, with a stronger association for pre-delivery BMI, suggesting that the maternal metabolic status at the end of gestation is more tightly connected with fetal exposure to kynurenine-pathway metabolites than baseline BMI alone [10]. Pre-delivery BMI may capture a more stable maternal metabolic phenotype, including residual adiposity, insulin resistance, and low-grade inflammation, all of which can influence placental substrate handling and enzyme activity. KYNA itself is biologically relevant because it is not merely an inert degradation product but an immunomodulatory and neuroactive metabolite, and increased KYNA may reflect diversion of TRP toward protective or adaptive pathways under metabolic stress [7]. In obesity, the kynurenine pathway is frequently upregulated; in the pregnancy setting, elevated cord KYNA may therefore be interpreted as a marker of altered placental metabolism in response to maternal adiposity rather than as a direct pathogenic mediator [22,23]. In our data, KYNA showed a positive correlation with birth weight but was not associated with Apgar scores or the presence of major pregnancy complications, which argues against a simple linear relationship between KYNA exposure and adverse short-term neonatal outcome.

### 4.4. Maternal QUIN and Cesarean Delivery

The pattern observed for maternal QUIN differed from that of TRP and KYNA. In multivariable models, maternal QUIN concentrations were higher in women undergoing intrapartum or emergency cesarean section and in those delivered by cesarean section for breech presentation, whereas QUIN did not differ meaningfully across most other clinical strata and was not related to neonatal condition at birth. Since cesarean delivery often clusters with pregnancy complications, fetal malpresentation, and altered obstetric risk profiles, the independent associations with intrapartum/emergency cesarean section and breech presentation suggest that QUIN may be capturing a broader obstetric phenotype rather than a delivery mode effect per se [10,15]. In addition, mode of delivery and intrapartum stress have been associated with distinct cord-blood kynurenine profiles in previous work, suggesting that operative delivery accompanies measurable metabolic differences. In our cohort, the specific association with breech-related cesarean section may be especially informative, because breech presentation frequently coexists with placental, fetal, or gestational factors that can lead to operative delivery. Therefore, elevated maternal QUIN is more appropriately interpreted as a marker of pregnancies with a more complex obstetric profile than as a direct determinant of cesarean section itself, particularly given the modest effect sizes and limited explained variance of the QUIN models [24,25].

### 4.5. Neonatal Condition and Tryptophan–Kynurenine Metabolites

The associations between maternal metabolites and early neonatal condition were weak and should be interpreted with caution. In univariable analyses, maternal QUIN and TRP showed inverse correlations with Apgar score at 1 min, and TRP also correlated inversely with Apgar at 5 min, but the correlation coefficients were small, and Apgar distributions were strongly skewed toward normal values. These findings are insufficient to support any causal interpretation and are better regarded as secondary signals that may reflect shared determinants of maternal metabolic state and transient neonatal adaptation rather than direct biochemical determinants of neonatal compromise. The absence of consistent relationships with Apgar scores in multivariable models is in line with previous observations that cord blood kynurenine metabolites are more strongly related to indices of fetal growth and later adiposity than to immediate perinatal adaptation. Correlations between kynurenine pathway metabolites and Apgar scores were weak and observed against a pronounced ceiling effect (median Apgar 10 at 1 min, range 6–10), making them susceptible to disproportionate influence from a small number of low-scoring outliers. These associations did not survive correction for multiple comparisons and should be regarded as a preliminary exploratory signal only, not as evidence of a biologically meaningful relationship. Replication in cohorts with a broader distribution of Apgar scores is needed before these associations can be considered informative.

### 4.6. Biological Interpretation

Taken together, our findings support the concept that the tryptophan–kynurenine pathway is a sensitive integrator of maternal and fetal physiology during pregnancy. The placenta is not just a passive conduit for metabolites; it actively regulates tryptophan transport and kynurenine metabolism, and changes in this pathway may influence immune tolerance, vascular adaptation, and fetal nutrient exposure. Within this framework, maternal TRP appears to be more closely related to gestational timing and intercurrent inflammatory burden, cord KYNA appears to be more strongly linked to maternal adiposity and fetal growth-related context, and maternal QUIN appears to reflect obstetric complexity and specific modes/indications of operative delivery. The absence of consistent associations with birth weight, Apgar scores or most maternal comorbidities suggests that these metabolites do not simply mirror overall pregnancy risk but rather respond to specific biological domains. These results are also consistent with the emerging view that cord blood kynurenine metabolites are associated with offspring adiposity and may represent early markers of metabolic programming, especially in the setting of maternal obesity and chronic low-grade inflammation. Because KYNA and QUIN were linked to maternal BMI and delivery characteristics in our study, they may capture both maternal metabolic health and pregnancy-specific adaptations of the placenta. This makes the pathway attractive as a source of candidate biomarkers for risk stratification in pregnancy, while at the same time underlining the need to account for gestational age, maternal metabolic status and obstetric context when interpreting tryptophan-related metabolites.

### 4.7. Study Limitations and Implications

From a translational perspective, these data add to the growing evidence that kynurenine pathway metabolites are not merely bystanders but potentially informative readouts of the maternal–fetal interface. The associations with gestational age, antibiotic exposure, BMI and cesarean delivery suggest that the pathway responds to multiple biological domains, including inflammation, metabolism and obstetric stress. This integrative behavior strengthens the rationale for longitudinal studies measuring the pathway across gestation, together with placental expression of key enzymes and transporters, and relating these profiles to both short- and long-term offspring outcomes. Future research should also explore whether composite metabolite signatures, rather than single metabolites, may provide better discrimination of clinically relevant pregnancy phenotypes.

### 4.8. Tryptophan Pathway Metabolites in Paired Maternal and Umbilical Cord Blood

The results of our study confirm a consistently reported finding in the literature, according to which the concentrations of tryptophan and its kynurenine pathway metabolites are significantly higher in umbilical cord blood than in maternal peripheral blood. This phenomenon was originally described by Kamimura et al. and Morita et al. [26,27], and has since been repeatedly corroborated over subsequent decades. In a systematic review by Broekhuizen et al. [20] published in the International Journal of Environmental Research and Public Health, data from numerous studies were compiled, collectively demonstrating that total TRP, KYN, KYNA, and QUIN concentrations are markedly higher in the fetal than in the maternal circulation at term. The elevation of TRP in umbilical cord blood observed in the present study is consistent with the data summarized by Broekhuizen et al. [20], in which TRP concentrations in the umbilical vein ranged from 60 to 101 µmol/L compared with 28–53 µmol/L in the maternal vein. These findings reflect active placental tryptophan transport, mediated primarily by the LAT1 (SLC7A5) and LAT2 (SLC7A8) transporters, rather than passive diffusion.

The nearly 2-fold elevation of the KYN/TRP ratio in umbilical cord blood relative to maternal blood observed in the present study is consistent with data from the GUSTO cohort reported by Tan et al. [13] where the KYN/TRP ratio in cord blood was approximately 4.6 × 10^−2^, compared with approximately 2.3 × 10^−2^ in maternal blood collected at 26–28 weeks of gestation. As noted by Broekhuizen et al. [20], however, the elevated KYN concentrations in the fetal circulation do not appear to result directly from placental IDO activity, since addition of TRP to the maternal circuit in ex vivo cotyledon perfusion models did not increase the release of kynurenine metabolites into the fetal circulation. This suggests that the high activity of the IDO/TDO pathway in the fetal compartment is largely autonomous, depending on endogenous metabolite production by fetal tissues or on active carrier-mediated transport.

The more than 4-fold elevation of the KYNA/KYN ratio in umbilical cord blood observed in our study warrants particular attention in the context of neuroprotection. In the GUSTO study, Tan et al. [13] reported KYNA concentrations in cord blood of approximately 350 nmol/L compared with approximately 18 nmol/L in maternal blood collected in the second trimester, a 19-fold difference. Such a dramatic enrichment of KYNA in the fetal compartment has been attributed to enhanced activity of kynurenine aminotransferases (KAT), particularly KAT-3, whose expression in the human placenta is markedly higher than that of KAT-2. Kynurenic acid is an endogenous broad-spectrum antagonist of ionotropic glutamate receptors, including NMDA receptors, and plays a pivotal role in protecting the developing fetal brain from glutamate-mediated excitotoxicity. The selective enrichment of KYNA in the fetal circulation may therefore represent an evolutionarily conserved mechanism of perinatal neuroprotection, whose disruption, for instance in the course of preeclampsia, gestational diabetes mellitus, or intrapartum hypoxia, could predispose the neonate to central nervous system injury.

The reduction in the QUIN/KYN ratio in umbilical cord blood relative to maternal blood observed in our study indicates that the neurotoxic branch of the kynurenine pathway, proceeding via kynurenine 3-monooxygenase (KMO) toward quinolinic acid, is relatively suppressed in the fetal compartment. These findings are consistent with data reported by Broekhuizen et al. and Murthi et al. [20,28], who demonstrated that KMO expression in the placenta is low under physiological conditions and further reduced in placentas from pregnancies complicated by fetal growth restriction (FGR). Notably, Kamimura et al. [26] observed that anthranilic acid, the product of the kynureninase branch, was the only metabolite that did not exhibit preferential enrichment in fetal blood.

The absence of significant Spearman correlations between maternal and umbilical cord blood concentrations of TRP, KYN, and KYNA in our study is in line with the ex vivo findings of Broekhuizen et al., suggesting that the placenta acts as an active regulator rather than a passive membrane with respect to kynurenine pathway metabolites. The weak but statistically significant correlation observed for QUIN alone may reflect a more passive, diffusion-dependent mechanism of placental transfer for quinolinic acid. This contrasts with the findings of Tan et al. [13] from the GUSTO cohort, in which all metabolites showed significant Pearson correlations between maternal and cord blood; this discrepancy may be attributable to differences in the timing of maternal blood collection (26–28 weeks of gestation in the GUSTO cohort versus the time of cesarean delivery in the present study), differences in analytical methodology, and population-specific characteristics.

## 5. Conclusions

In this cross-sectional cohort of women delivering by cesarean section, maternal and fetal tryptophan metabolites appeared to show distinct, albeit relatively modest, associations with gestational timing, maternal adiposity, indicators of antibiotic exposure during pregnancy, and selected obstetric indications for operative delivery. Maternal TRP tended to be inversely associated with gestational age and was higher in women who received antibiotic treatment during pregnancy, whereas cord blood KYNA was positively associated with maternal BMI, particularly pre-delivery BMI, which may suggest that certain aspects of maternal metabolic status are reflected in fetal exposure to tryptophan catabolism pathway metabolites. Maternal QUIN concentrations were also higher in women undergoing intrapartum/emergency cesarean section and in those delivered for breech presentation, which may indicate a relationship with more complex obstetric phenotypes rather than with neonatal status directly. At the same time, the explanatory power of the models was limited, and most pregnancy complications and neonatal outcomes, including birth weight and Apgar scores, were not clearly associated with the measured metabolites. These findings therefore should be interpreted with caution and viewed primarily as hypothesis-generating. Overall, the present study suggests that the placental tryptophan catabolism pathway may offer complementary insight into the maternal metabolic and obstetric milieu, although these observations require confirmation in larger, longitudinal studies including pregnancies ending in vaginal birth and more detailed characterization of inflammatory and metabolic status. The present study demonstrates also that all major tryptophan catabolites—TRP, KYN, KYNA, and QUIN—are significantly more concentrated in umbilical cord blood than in simultaneously obtained maternal peripheral blood at the time of cesarean delivery, with the most pronounced enrichment observed for KYNA (~15-fold). The observed ratio pattern in cord blood, elevated KYN/TRP and KYNA/KYN alongside a relatively reduced QUIN/KYN, suggests a differential distribution of kynurenine pathway flux between the maternal and fetal compartments at term. Whether this reflects a preferential channelling toward the cytoprotective KYNA branch at the expense of the neurotoxic quinolinate pathway, or simply differential placental transport and metabolic activity, cannot be determined from the present cross-sectional data.

All findings should therefore be interpreted with caution and regarded strictly as hypothesis-generating, exploratory signals. The study additionally confirms that all four major tryptophan–kynurenine metabolites (TRP, KYN, KYNA, and QUIN) are significantly more concentrated in umbilical cord blood than in simultaneously obtained maternal peripheral blood at term, consistent with the established literature on active placental tryptophan transport and fetal metabolic autonomy. Taken together, these observations suggest that the tryptophan–kynurenine pathway may offer complementary insight into maternal metabolic and obstetric context, but confirmatory evidence from larger, prospectively designed, longitudinal studies, including populations delivering vaginally and employing LC-MS/MS-based metabolite quantification, is needed before any clinical interpretation can be considered. All associations reported here, including those depicted in Figure 3, are based on post hoc, uncorrected comparisons in a modestly sized cohort, and none survived correction for multiple testing. They require replication in independent, adequately powered cohorts before any causal or clinical interpretation is warranted.

## 6. Limitations

This study has several limitations that should be considered when interpreting the findings. Firstly, its cross-sectional, single-center design and the relatively small sample size of women delivering exclusively by cesarean section limit statistical power and generalizability to the broader obstetric population, including vaginal births. Secondly, tryptophan-pathway metabolites were measured once, at term, using ELISA-based assays; thus, temporal changes across gestation and potential preanalytical variability could not be assessed. This study used commercial ELISA kits rather than LC-MS/MS, the current reference method for kynurenine pathway metabolites. ELISA is subject to lower analytical specificity and potential cross-reactivity among structurally related metabolites. Full in-matrix analytical validation (dilution linearity, recovery/spike-in, duplicate measurement) was not performed. LC-MS/MS was not available in our laboratory setting at the time of the study. Consequently, absolute concentration values reported here should not be directly compared to those obtained by chromatographic methods in other studies; only relative patterns and directions of association should be considered for cross-study comparison. Thirdly, detailed data on the type and severity of infections, concomitant medications, dietary intake, and other potential modifiers of tryptophan metabolism were not available, raising the possibility of residual confounding. Fourthly, the exact timing of maternal venous blood sampling relative to the onset of labor, administration of antibiotic prophylaxis, administration of anesthesia, and the clinical decision to proceed with an emergency or intrapartum cesarean section was not recorded. This represents an important limitation for the interpretation of elevated maternal QUIN levels in the intrapartum/emergency subgroup, as it is not possible to determine whether observed differences reflect the underlying obstetric complication, the physiological stress of labor, pharmacological exposures, or some combination thereof. This study was designed as an exploratory pilot investigation. The sample size was determined by the number of eligible women recruited during the study period. Given the exploratory nature of the study and the large number of statistical comparisons performed, the possibility of type I error cannot be excluded since no association presented in Table 4, Table 5, Table 6, Table 7 and Table 8 remained statistically significant after Bonferroni correction. Therefore, all findings should be interpreted as hypothesis-generating and require confirmation in larger cohorts. WHO BMI thresholds were originally developed for the general adult population and have not been formally validated for classification of BMI measured at term pregnancy or in the immediate peripartum period, which includes fetal, placental, and fluid-related mass. Their application here to pre-delivery BMI should be regarded as a pragmatic approximation rather than a clinically validated categorization. Finally, the regression models explained only a modest proportion of variance in metabolite concentrations, so the observed associations should be regarded as hypothesis-generating rather than definitive evidence of causal relationships.

Enrollment of patients undergoing emergency or intrapartum cesarean delivery required that the patient’s clinical condition permit the informed consent process. This likely introduced a selection bias whereby the most clinically unstable patients—who may have exhibited the greatest degree of physiological and oxidative stress, and potentially the highest QUIN concentrations were systematically excluded from the emergency/intrapartum subgroup. As a result, the observed difference in maternal QUIN between elective and emergency/intrapartum cesarean deliveries may underestimate the true difference. Combined with the small size of the emergency/intrapartum subgroup and the fact that this comparison did not survive FDR correction, this comparison should be interpreted with considerable caution and regarded as a preliminary signal warranting further investigation in studies with alternative consent frameworks (e.g., deferred or proxy consent) that would allow inclusion of the most severely affected patients.

## Figures and Tables

**Table 1:** Maternal demographic and anthropometric characteristics (n = 73).

Variable	Mean ± SD	Median	Range (Min–Max)	Q1–Q3
Maternal age (years)	31.91 ± 4.40	32.32	21.64–42.09	28.76–34.50
Height (m)	1.67 ± 0.06	1.66	1.57–1.88	1.63–1.71
Pre-pregnancy weight (self-reported) (kg)	79.20 ± 20.79	74.70	47.00–129.00	61.25–93.30
Pre-pregnancy BMI (kg/m^2^)	28.18 ± 6.92	26.78	18.36–47.82	22.73–32.42
Pre-delivery weight (measured at hospital admission) (kg)	92.04 ± 19.82	89.50	60.00–141.00	75.10–104.88
Pre-delivery BMI (kg/m^2^)	32.78 ± 6.44	31.91	23.14–53.73	27.52–37.02
Gestational age at delivery (weeks)	38.89 ± 0.96	39.00	36.14–41.00	38.36–39.14
Previous cesarean sections (number)	0.64 ± 0.71	1.00	0–3	0–1

Data are presented as mean ± standard deviation (SD), median, range, and interquartile range (Q1–Q3).

**Table 2:** The most frequent indications for cesarean section in the study cohort are presented as absolute numbers and percentages of all (n = 73).

Indication for Cesarean Section	n	%
Cephalopelvic disproportion	15	20.5%
Fetal distress	12	16.4%
Tokophobia (maternal request)	9	12.3%
Breech presentation	8	11.0%
Ophthalmologic indications	5	6.8%
Orthopedic indications	5	6.8%
Other	19	26.0%
Total	73	100%

**Table 3:** Maternal and umbilical tryptophan pathway metabolites (n = 73).

	Variable	Mean ± SD	Median	Range (Min–Max)	Q1–Q3
**Maternal (n = 73)**	QUIN (ng/mL)	47.85 ± 15.18	44.89	12.08–91.47	38.38–56.57
	TRP (µg/mL)	5487.17 ± 1397.97	5336.99	2126.35–9387.39	4533.27–6390.61
	KYNA (nmol/L)	4.27 ± 1.49	3.95	1.70–8.82	3.36–5.03
	KYN (pmol/mL)	285.33 ± 112.17	254.10	83.08–581.07	208.52–337.02
**Umbilical (n = 73)**	QUIN (ng/mL)	130.30 ± 31.21	125.53	15.40–232.52	111.00–142.02
	TRP (µg/mL)	11,738.47 ± 2302.51	11,571.54	5528–19,219.71	10,190.78–13,007.77
	KYNA (nmol/L)	63.36 ± 16.54	61.68	20.46–109.71	51.38–76.43
	KYN (pmol/mL)	1025.07 ± 240.58	995.80	316.01–1827.00	923.24–1133.65

**Table 4:** Statistically significant differences in tryptophan-pathway metabolite concentrations between clinically defined subgroups (n = 73).

Clinical Comparison	Metabolite (Matrix)	Group	n	Mean ± SD	Median (Min–Max)	*p*-Value (Mann–Whitney U Test)
**Elective vs. intrapartum/emergency cesarean section**	Maternal QUIN (ng/mL)	Elective cesarean section	63	46.5 ± 15.3	44.4 (12.1–91.5)	0.038
		Intrapartum/emergency cesarean section	10	56.1 ± 11.7	55.2 (39.2–74.1)	
**Pre-delivery overweight/obesity (yes vs. no)**	Umbilical KYNA (ng/mL)	No postpartum overweight/obesity	50	59.9 ± 16.6	57.6 (20.5–109.7)	0.016
		Postpartum overweight/obesity	23	69.3 ± 15.0	76.0 (45.0–90.3)	

Only metabolite comparisons with *p* < 0.05 are presented; for all other clinical subgrouping variables (gestational diabetes, hypertension, thyroid dysfunction, antibiotic treatment, indications for cesarean section), no significant differences in metabolite concentrations were observed (all *p* ≥ 0.05).

**Table 5:** Significant Spearman rank correlations between clinical parameters and maternal and umbilical tryptophan-pathway metabolites (n = 73).

	Maternal				Umbilical			
**Clinical parameter**	QUIN	TRP	KYNA	KYN	QUIN	TRP	KYNA	KYN
**Birth weight (g)**	ρ = −0.126 *p* = 0.290	ρ = 0.076 *p* = 0.524	ρ = −0.036 *p* = 0.761	ρ = −0.038 *p* = 0.747	ρ = 0.124 *p* = 0.296	ρ = 0.059 *p* = 0.621	ρ = 0.28 * p = 0.018	ρ = 0.090 *p* = 0.449
**Apgar score at 1 min**	ρ = −0.26 * p = 0.028	ρ = −0.26 * p = 0.023	ρ = −0.145 *p* = 0.222	ρ = −0.126 *p* = 0.288	ρ = 0.026 *p* = 0.828	ρ = 0.114 *p* = 0.337	ρ = 0.086 *p* = 0.468	ρ = 0.001 *p* = 0.992
**Apgar score at 5 min**	ρ = −0.084 *p* = 0.482	ρ = −0.24 * p = 0.044	ρ = −0.142 *p* = 0.231	ρ = −0.101 *p* = 0.135	ρ = −0.072 *p* = 0.543	ρ = 0.133 *p* = 0.260	ρ = 0.016 *p* = 0.893	ρ = 0.014 *p* = 0.907
**Postpartum BMI (kg/m^2^)**	ρ = −0.012 *p* = 0.918	ρ = −0.025 *p* = 0.834	ρ = 0.095 *p* = 0.425	ρ = 0.024 *p* = 0.839	ρ = −0.041 *p* = 0.732	ρ = 0.048 *p* = 0.684	ρ = 0.29 * p = 0.014	ρ = 0.030 *p* = 0.789

Spearman’s rank correlation coefficient (ρ); *p*-value (*p*); *—significant Spearman rank.

**Table 6:** Univariate linear regression model for umbilical kynurenic acid concentration.

Predictor	β (Unstandardized)	SE	Standardized β	t	*p*-Value	95% CI for β
**Intercept**	38.1	9.7		3.93	<0.001	18.8 to 57.5
**Pre-delivery BMI (kg/m^2^)**	0.77	0.29	0.30	2.65	0.010	0.19 to 1.35

Predictor—independent variable; β (unstandardized)—unstandardized regression coefficient; SE—standard error of the coefficient; Standardized β—standardized regression coefficient (beta); t—t statistic for testing β = 0; *p*-value—probability value for the *t* test; 95% CI for β—95% confidence interval for unstandardized regression coefficient.

**Table 7:** Multivariable linear regression model for maternal tryptophan concentration.

Predictor	β (Unstandardized)	SE	Standardized β	t	*p*-Value	95% CI for β
**Intercept**	20,402.0	6221.8		3.28	0.002	7989.9 to 32,814.2
**Gestational age at delivery (weeks)**	−294.1	161.6	−0.20	−1.82	0.073	−616.4 to 28.2
**Antibiotic treatment during pregnancy (yes vs. no)**	962.9	398.7	0.27	2.42	0.018	167.6 to 1758.2
**Apgar score at 1 min**	−381.1	191.3	−0.22	−1.99	0.050	−762.7 to 0.6

Predictor—independent variable; β (unstandardized)—unstandardized regression coefficient; SE—standard error of the coefficient; Standardized β—standardized regression coefficient (beta); t—t statistic for testing β = 0; *p*-value—probability value for the *t* test; 95% CI for β—95% confidence interval for unstandardized regression coefficient.

**Table 8:** Multivariable linear regression model for maternal quinolinic acid concentration according to indications for cesarean section (n = 73).

Predictor	β (Unstandardized)	SE	Standardized β	t	*p*-Value	95% CI for β
**Intercept**	44.79	1.91		23.49	<0.001	40.98 to 48.59
**Intrapartum/emergency cesarean section (vs. elective)**	11.36	4.90	0.26	2.32	0.023	1.59 to 21.13
**Breech presentation as obstetric indication (yes vs. no)**	15.73	5.72	0.31	2.75	0.008	4.32 to 27.14

Predictor—independent variable; β (unstandardized)—unstandardized regression coefficient; SE—standard error of the coefficient; Standardized β—standardized regression coefficient (beta); t—t statistic for testing β = 0; *p*-value—probability value for the *t* test; 95% CI for β—95% confidence interval for unstandardized regression coefficient.

**Table 9:** Paired comparison of tryptophan, kynurenine, kynurenic acid, and quinolinic acid concentrations and their metabolic ratios (KYN/TRP, KYNA/KYN, QUIN/KYN) in maternal and umbilical cord blood: results of Wilcoxon signed-rank test and Spearman correlation analysis.

Parameter	N	Maternal Median [IQR]	Cord Median [IQR]	*p* (Wilcoxon)	Cord/Mat Ratio Median [IQR]
**TRP (ng/mL)**	73	5338 [4562–6435]	11,606 [10,231–13,008]	<0.0001	-
**KYN (ng/mL)**	73	254 [212–341]	998 [923–1137]	<0.0001	-
**KYNA (ng/mL)**	73	3.97 [3.44–5.08]	61.92 [51.54–76.56]	<0.0001	-
**QUIN (ng/mL)**	73	44.90 [38.77–56.59]	125.71 [112.35–142.57]	<0.0001	-
**KYN/TRP × 10^3^**	73	45.50 [39.04–61.93]	85.12 [75.36–97.85]	-	1.82 [1.41–2.09]
**KYNA/KYN × 10^3^**	73	16.44 [12.09–20.58]	64.74 [53.34–74.05]	-	4.20 [2.87–5.31]
**QUIN/KYN**	73	0.177 [0.136–0.228]	0.122 [0.107–0.147]	-	0.76 [0.55–0.97]

## Data Availability

Data are available on request from the first author.

## Supplementary Materials

The following supporting information can be downloaded at: https://www.mdpi.com/article/10.3390/life16071166/s1, Table S1: Comparison of variables.
